# Organic/Inorganic-Based Flexible Membrane for a Room-Temperature Electronic Gas Sensor

**DOI:** 10.3390/nano12122037

**Published:** 2022-06-14

**Authors:** Husam H. D. AlTakroori, Ashraf Ali, Yaser E. Greish, Naser Qamhieh, Saleh T. Mahmoud

**Affiliations:** 1 Department of Physics, United Arab Emirates University, Al-Ain 15551, United Arab Emirates; 202070073@uaeu.ac.ae (H.H.D.A.); ashraf_ali@uaeu.ac.ae (A.A.); nqamhieh@uaeu.ac.ae (N.Q.); 2 Department of Chemistry, United Arab Emirates University, Al-Ain 15551, United Arab Emirates; y.afifi@uaeu.ac.ae; 3 Department of Ceramics, National Research Centre, Cairo 68824, Egypt

**Keywords:** organic polymer, H_2_S sensor, metal oxide semiconductor, ZnO NPs, organic-inorganic nanocomposite

## Abstract

A room temperature (RT) H_2_S gas sensor based on organic–inorganic nanocomposites has been developed by incorporating zinc oxide (ZnO) nanoparticles (NPs) into a conductivity-controlled organic polymer matrix. A homogeneous solution containing poly (vinyl alcohol) (PVA) and ionic liquid (IL) and further doped with ZnO NPs was used for the fabrication of a flexible membrane (approx. 200 μm in thickness). The sensor was assessed for its performance against hazardous gases at RT (23 °C). The obtained sensor exhibited good sensitivity, with a detection limit of 15 ppm, and a fast time response (24 ± 3 s) toward H_2_S gas. The sensor also showed excellent repeatability, long-term stability and selectivity toward H_2_S gas among other test gases. Furthermore, the sensor depicted a high flexibility, low cost, easy fabrication and low power consumption, thus holding great promise for flexible electronic gas sensors.

## 1. Introduction

Nature has been so much stained and strained by the actions of humans, resulting in a rapid increase in environmental pollution. Therefore, its monitoring has become the need of the hour and a priority for the health of mankind. One of the threats that make our environment hostile is hazardous gases. Some gases are more toxic than others, depending on their effects that range from mild respiratory symptoms to eventual death. Enormous efforts have been invested in tackling this situation by developing sensors, which are highly sensitive, selective and stable toward the target gases, among others [1,2,3,4].

Occupational exposure to toxic gases has been a major concern in the petroleum industry, with hydrogen sulfide (H_2_S) being one of the contenders. It is also known as a “knock down gas”, as exposure to it may lead to immediate loss of consciousness and death [5,6,7]. Overlong exposure to 10–500 parts per million (ppm) causes respiratory symptoms, and exposure to 500–1000 ppm can be fatal [5]. In addition to the petroleum industry, H_2_S gas is evolved in agricultural industry, garbage disposal sites, sewers, tanneries, wastewater treatment and mining industries, to name a few [8,9,10,11].

Recently, there have been numerous developments in the area of gas detection systems. The sensors exploit various principles, such as chemiresistive sensing [12], capacitive-based sensing [13], quartz crystal microbalance sensors [14], optical transduction [15] and organic field-effect transistor sensors. Among these, chemiresistive sensors that track the change in electrical conductivity or resistance of the sensing material have attracted much attention due to the high sensitivity, low cost, ease of fabrication and possibility of miniaturization. 

Zinc oxide (ZnO) possesses a range of physicochemical properties that are apt for hazardous gas sensing applications. It is classified as an n-type II-VI semiconductor with a wide bandgap (3.37 eV), a large excitation binding energy (60 meV) and high electron mobility (400 cm^2^V^−1^s^−1^) [16,17]. Additionally, ZnO is chemically stable, environmentally friendly and can be synthesized at a low cost [18], making it a promising material for the task. The crystalline nature of ZnO allows it to be grown in different structures, such as nanoparticles (0D), one-dimensional (1D), two-dimensional (2D) and three-dimensional (3D) structures [19]. The morphologies, such as nanorods, wires, needles, spheres, ellipsoids and flowers [20], enable controlling the surface area-to-volume ratio, thereby enhancing the utility of the material in a plethora of sensing applications. In addition to that, the bandgap of the material can be altered by doping ZnO with various materials, which improve the sensitivity of the sensor with varying operating temperatures [21,22,23,24,25,26,27]. 

The working principle of the ZnO-based sensors is usually evaluated at elevated temperatures of about 300–500 °C [28,29,30,31,32]. To attain these temperatures, external energy has to be provided to the sensor, which would mean an increase in the operational cost. Furthermore, at these temperatures, the flammable and explosive gases are more prone to explosions due to their low ignition point. Additionally, at higher temperatures, the stability of the material reduces, which would generate inaccurate results [33,34], and this also leads to shortening the life time of the sensor. The sensors that are developed to operate at room temperature do not require any additional heating elements; hence, their operational cost is reduced. In addition, they can be made portable, thus their risk of explosion is avoided.

Organic polymers, such as PVA, are characterized by their wide abundance and their known promising properties, such as flexibility, environmental friendliness, thermal stability and the ability to form electrolytes by virtue of their hydrophilic nature [35]. Moreover, the conductivity of PVA can be controlled by doping it with an ionic liquid (IL), such as glycerol [36,37,38]. These combined properties can be further exploited by doping the polymer matrix with materials that have an affinity toward the detection of H_2_S gas, so that changes in their conductivity or resistivity can be recorded. 

The aim of this investigation is to develop an organic–inorganic hybrid gas sensor, where a matrix of PVA/IL polymer is doped with ZnO NPs, and to explore its gas sensing capabilities toward H_2_S gas, among other hazardous gases. The low fabrication and operational costs, flexibility and operational ability at room temperature enable the device to be deployed for monitoring these threats in real-time application scenarios.

## 2. Materials and Methods

### 2.1. Materials

Zinc oxide (ZnO) nano-powder (<100 nm), poly (vinyl alcohol) (PVA) (MW ~ 61,000 Da) and glycerol (>99.5%) were purchased from Sigma-Aldrich (St. Louis, MO, USA) and used without further purification.

### 2.2. Fabrication of the ZnO/PVA/IL Membrane

**Figure 1:**
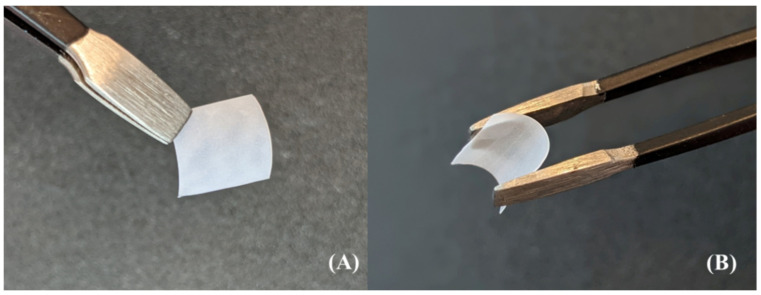
(**A**). 1 × 1 cm^2^ sample of ZnO/PVA/IL membrane. (**B**) Demonstration of its high flexibility.

An amount of 50 mg of ZnO NPs was first dispersed in a 40 mL of distilled water (DW) using a vortex shaker. Then, 1000 mg of PVA granules was added to the dispersed NPs and stirred vigorously at 90 °C until a homogenous solution was obtained. After that, the mixture was doped with 1 mL of glycerol and was kept under continuous stirring for 20 min. The mixture formed was then cast onto a Petri dish and was dried in air at 70 °C for 18 h, resulting in a flexible membrane, as shown in Figure 1. The thickness of the membrane was determined to be 200 μm.

### 2.3. Characterization

ZnO NPs were subjected to analysis for their structural and morphological characteristics. A powder X-ray diffraction (PXRD) of the as-received ZnO NPs was performed using a Rigaku, MiniFlex 600-C instrument, Austin, TX, USA, with a Cu Ka X-ray with a scan range of 2–80° and at a scan rate of 1°/min. The morphology of the ZnO powder, as well as the membrane, was investigated using a Thermoscientific Quattro S SEM instrument (Waltham, MA, USA) at an operating voltage of 15 kV. The membrane was further analyzed for its composition by Fourier transform infrared (FT-IR) spectroscopy using a Thermo Nicolet, NEXUS, 470 FT-IR instrument. A KBr disk method over a scan range of 400–4000 cm^−1^ was used. The TGA plots were recorded using TGA-Q500, TA Instruments, New Castle, DE, USA, with the ramp rate of 20 °C/min and within a temperature range of 30–800 °C.

### 2.4. Sensor Fabrication and H_2_S Gas Sensing Tests

The sensor was fabricated as delineated in our previous work [36]. A mixture of the test gas, diluted with synthetic air, was introduced into the chamber using Bronkhorst mass flow controllers (MFC). The test chamber was sealed and placed inside a fume hood to maintain a non-humid atmosphere throughout the measurement. The device was kept at RT throughout the testing sequences. Keithley Instruments source measurement unit (KI236) was used to record the response of the sensor, applying a constant bias voltage of 4 V between the electrodes. Labview software (version 21.0.1–32-bit) was used to interface and record the response of these units.

## 3. Results and Discussion

### 3.1. Structural and Morphological Characterization of ZnO NPs and ZnO/PVA/IL Membrane

**Figure 2:**
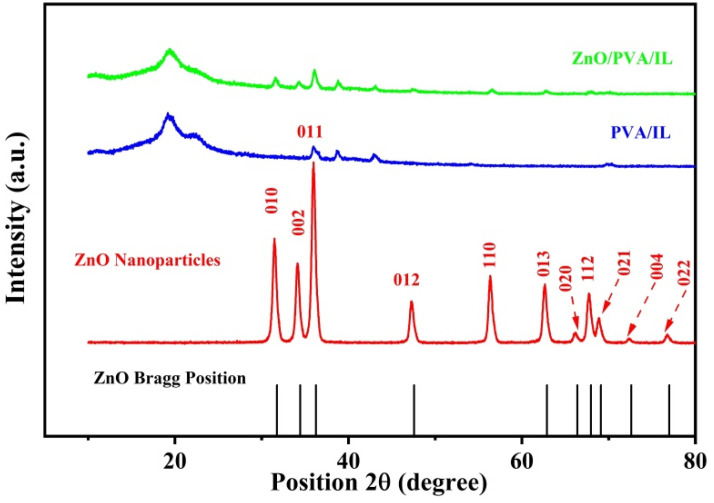
Comparison of XRD pattern for ZnO NPs, PVA/IL membrane and ZnO/PVA/IL composite membrane.

Figure 2 shows a comparison between the PXRD patterns of the as-received ZnO NPs, the PVA/IL membrane and the ZnO-doped PVA/IL membrane. Compared with the JCPDS PXRD pattern of pure ZnO (Card # 36-1451), the as-received ZnO shows a phase-pure composition where all peaks of the standard ZnO were observed. The weakly crystalline PVA within the PVA/IL and composite matrices showed two broad peaks at 19.3 and 20.8° [39,40,41,42]. Additionally, the composite membrane confirmed the presence of the ZnO NPs, where its characteristic peaks representing the 010, 002 and 011 planes were observed.

**Figure 3:**
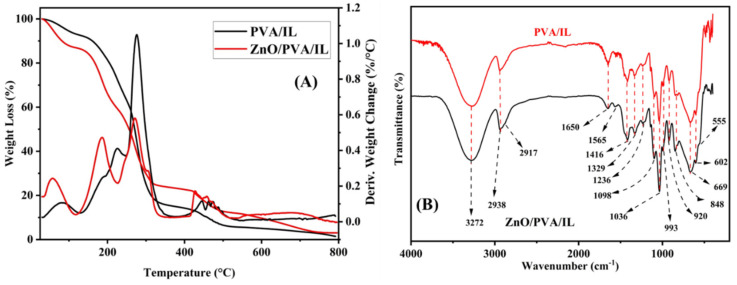
(**A**) TGA comparison of PVA/IL and ZnO/PVA/IL membranes. (**B**) FTIR spectra of PVA/IL and ZnO/PVA/IL membrane.

The membrane was subjected to thermogravimetric analysis (TGA), as seen in Figure 3A. From the decomposition curves, we are able to observe that there is a gradual loss in weight due to the evaporation of water molecules and adsorbed moisture content. Additionally, the loss in weight between 300 and 550 °C is attributed to the removal of organic groups [41]. No appreciable weight loss beyond 700 °C was observed, inferring the formation of stable inorganic phases [43]. A slight variation in the thermal profile of the composite membrane was observed, which is due to the fact that ZnO NPs are thermally stable throughout the heating cycle. The recorded profile matches well with the reported literature [41], confirming the incorporation of ZnO NPs into the polymer matrix.

The FTIR analysis from Figure 3B shows that the membrane has the functional peaks associated with PVA [42,44] and ZnO NPs [42,45], along with a slight shift in their positions, inferring the incorporation of the NPs in the polymer matrix. The broad absorption bands at 3272 cm^−1^ and 2938 cm^−1^ denoting the O-H and C-H stretching modes, respectively, are observed. The band at 555 cm^−1^ is attributed to the Zn-O stretching vibration mode [42], while the bands at 1142 cm^−1^ and 1329 cm^−1^ are ascribed to the primary and secondary alcohol in-plane bending modes [45]. 

**Figure 4:**
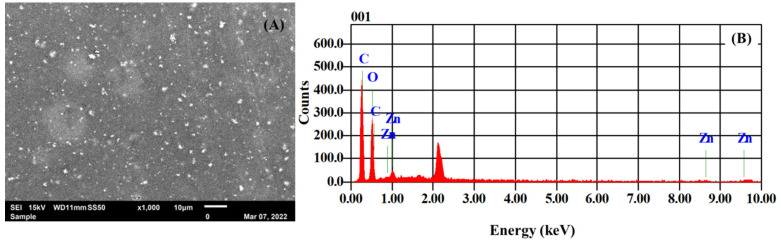
(**A**). SEM image of ZnO/PVA/IL membrane. (**B**) EDX spectrum of ZnO/PVA/IL membrane.

Figure 4 shows the SEM and EDX analysis, respectively, of the ZnO/PVA/IL membrane. The image shows a uniform dispersion of the NPs incorporated into the membrane. However, ZnO NPs were observed in the form of agglomerates with a uniform size distribution, which is a common criterion of NPs dispersed in highly viscous PVA solutions. The elemental analysis confirms the presence of Zn and O in the composite membrane.

### 3.2. Gas Sensing Performance

**Figure 5:**
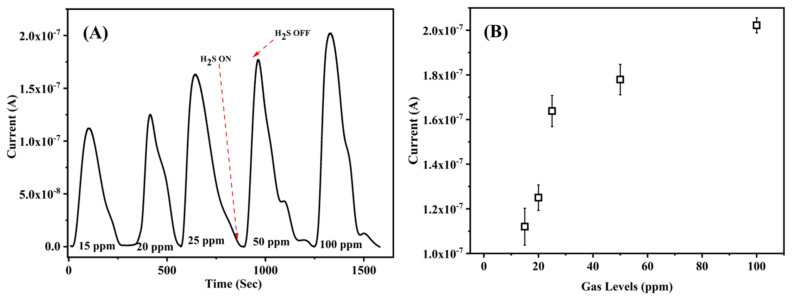
(**A**) Current response of ZnO/PVA/IL membrane to H_2_S gas exposure at different concentrations with respect to time. (**B**) The current response of the membrane as a function of gas concentrations.

The composite membrane sensor was evaluated for its response against H_2_S gas exposure at different concentrations with respect to time at RT. Figure 5A shows the sensor response to different concentrations of H_2_S gas over a period of time. Figure 5B shows the current response with respect to the test gas concentration. The sensor response (S%) was calculated using Equation (1).

(1) $$S\;\left(\%\right)=\frac{I_{g}-I_{a}}{I_{a}}\times 100$$

where *I_a_* is the reference current when the sensor was exposed to air, and *I_g_* is the current measured when the sensor was exposed to H_2_S.

An increase in the response was observed as a result of increasing the concentration of the test gas. After each exposure to the test gas, the chamber was flushed with synthetic air to remove any residual gas molecules. It is also noted that while the chamber was flushed, the current values reduced to their starting values in the absence of the test gas, demonstrating the reversibility of the sensor.

**Figure 6:**
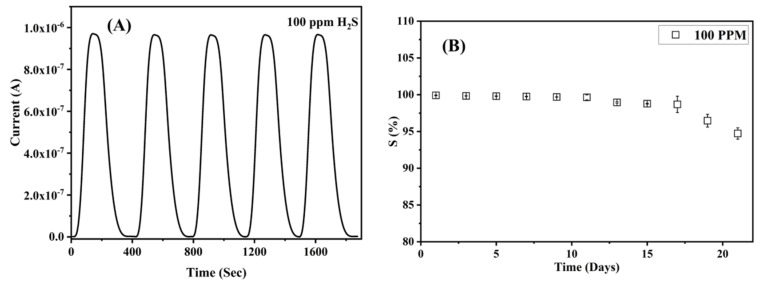
(**A**) Repeatability of the ZnO/PVA/IL membrane at 100 ppm H_2_S. (**B**) Long-term stability of the ZnO/PVA/IL membrane for 21 days at 100 ppm H_2_S gas.

The sensor was further tested for its repeatability and long-term stability. Figure 6A demonstrates an excellent repeatability of sensing in the membrane with a near-identical current response for five cycles of exposure to H_2_S gas at a concentration of 100 ppm. Figure 6B shows the response of the membrane to 100 ppm of H_2_S gas exposed for 21 days, which demonstrates its long-term stability. It can be seen that the response is in the 94–99% region, with minimal error bars.

Furthermore, the sensor’s response time, which is the time needed for the sensor to reach 90% of its maximum response, was also calculated. The average time response for the proposed sensor was 24 ± 3 s for 100 ppm of H_2_S gas at RT. Another parameter that would evaluate the sensor’s performance would be the recovery time. The recovery time of the sensor is defined as the time required by the sensor to recover to 90% of its baseline signal after the target gas has been stopped. The average time for the sensor was calculated to be 112 ± 5 s for 100 ppm of H_2_S gas at RT. Table 1 outlines a comparison of the performance of the proposed sensor with the reported literature.

**Figure 7:**
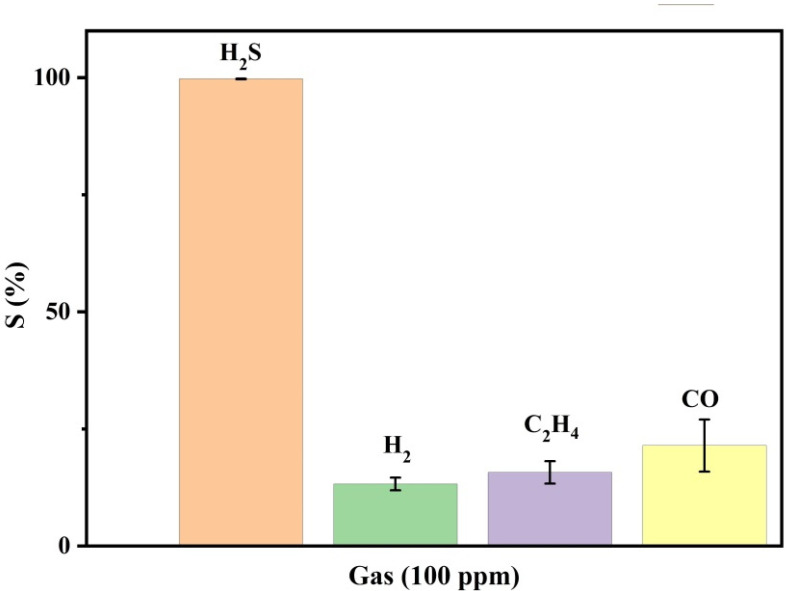
Selectivity of the ZnO/PVA/IL membrane to 100 ppm of H_2_S compared to H_2_, C_2_H_4_, CO gases at 100 ppm.

Finally, the sensor’s selectivity was measured by exposing it to 100 ppm of H_2_, C_2_H_4_ and CO gases at RT. Figure 7 illustrates the response of the sensor to those gases, showing a significant difference between the response to H_2_S compared to the other gases, which indicates an excellent selectivity of the proposed sensor toward those gases.

### 3.3. Gas Sensing Mechanism

**Figure 8:**
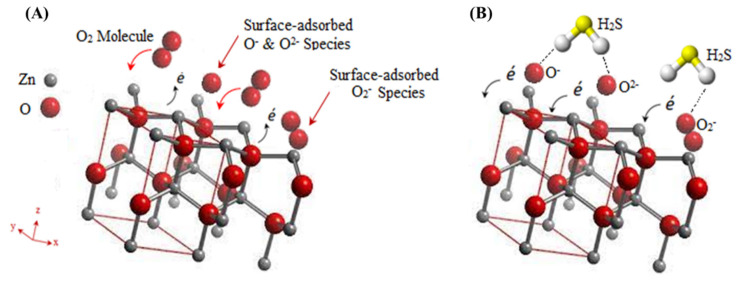
Mechanism of interaction between ZnO crystal lattice and O_2_ molecules in air (**A**) and when flushed with H_2_S gas (**B**).

The sensing mechanism of the ZnO/PVA/IL can be elaborated as a surface sensing mechanism [16,55,56,57], which is based on a process of adsorption–oxidation–desorption. ZnO has a non-centrosymmetric wurtzite crystal structure with polar surfaces, as shown in Figure 8. When ZnO NPs, which are homogeneously embedded in a polymeric matrix, are flushed with synthetic air, the hydrophilic nature of the matrix allows the oxygen molecules to become adsorbed on the surface, which captures the electrons from the ZnO conduction band, leading to the formation of negatively charged ions (O_2_^−^, O^−^, O^2−^) [16] and thereby providing surface acceptors sites, as shown in Figure 8A.

Upon reaching equilibrium, the conductivity of the ZnO NPs reaches a steady state. Upon the exposure to H_2_S, as a reducing gas, a charge transfer process is initiated via the oxidization of the surface-adsorbed oxygen anions, providing additional free electrons that migrate into the conduction band of ZnO, as shown in Figure 8B. This process results in a reduction in the thickness of the depletion layer, which gradually reaches equilibrium; hence, the conductivity of the membrane is enhanced. When the chamber is flushed with synthetic air, the target gas molecules are gradually expelled from the membrane, which reduces the conductivity of the material to the base values recorded. The continuity of these steps explains the sensing mechanism of ZnO NPs, as depicted by our findings. 

The homogeneous distribution of the ZnO NPs within a polymeric matrix that contains a conductive IL is believed to provide an enhanced venue for the above-mentioned mechanism to take place at RT. This can be explained by the extensive H-bonding network that takes place between the -OH groups of the PVA polymeric matrix, as well as the IL. This network facilitates charge transfer across the composite membrane, following the adsorption of the oxygen species from air and their reduction by H_2_S in a steam of the latter gas [58]. At temperatures between 150 and 300 °C, a hybrid mechanism of surface reactivity and gas diffusion contributes to the sensitivity of the membrane, whereas above 300 °C, the sensitivity is limited to the gas diffusion phenomenon. Detailed equations representing the surface adsorption reactions were outlined by Kang et al. [16]. The sensitivity of the membrane reported in the current investigation would be supported by the former. Moreover, the even distribution of the ZnO NPs in the polymeric membrane would also support the enhancement of the sensitivity toward H_2_S gas in comparison to the membranes deprived of the NPs.

## 4. Conclusions

This investigation demonstrates the potential of fabricating high-performance H_2_S gas sensors based on organic–inorganic nanocomposites. The flexible mixed-matrix membrane was prepared by doping inorganic ZnO NPs into an organic PVA polymer together with IL. The fabricated membrane was investigated for its gas-sensing performance. The ZnO-free PVA/IL membrane did not show any sensing response toward H_2_S gas, whereas the ZnO-doped membrane showed good sensitivity, with a detection limit of 15 ppm and fast time response of 24 ± 3 s at RT, along with its high selectivity and long-term stability. Considering this low operating temperature, the requirement for external heating elements is not necessary, hence, the fabrication and operational costs are reduced. Moreover, the composites of the membrane are known to cause no harm to the environment, which makes them eco-friendly. Therefore, the proposed sensor could be implanted into electronic devices for potentially monitoring harmful gases in real time with a high efficiency.

## Figures and Tables

**Table 1:** A comparison of the sensor’s performance with the reported literature.

Sensor/Material	Gas	Operating Temp °C	Detection Limit (ppm)	Response Value	Response Time/Recovery Time (Second)	Ref.
ZnO/PVA/IL	H_2_S	RT	15	99%	24/112	This Work
Colloidal ZnO QDs	H_2_S	RT	50	113.5	16/820	[19]
ZnO/γ Fe_2_O_3_ Electrochemical	H_2_S	RT	250	80%	60/300	[46]
Al-ZnO spray pyrolysis	H_2_S	200	150	12.41%	200/209	[47]
Self-assembled polyaniline nanocapsules/ZnO hexagonal microdiscs	H_2_S	RT	50	11.5%	63/12	[48]
Lettuce like ZnO 3D	H_2_S	150	100	113	15/90	[49]
Cu-doped ZnO RGO	H_2_S	RT	100	0.87%	14/32	[50]
ZnO ZnS Heterostructure	H_2_S	150	5	0.88	N R	[51]
ZnO CuO composite	H_2_S	40	10	393.35	173/179	[52]
ZnO NPs	CO	RT	25	6%	N R	[53]
Dumbbell-shaped ZnO 3D	H_2_	60	100	20%	20/10	[54]

N R—Not Reported.

## Data Availability

Not applicable.

## References

[1] Joshi N., Hayasaka T., Liu Y., Liu H., Oliveira O.N., Lin L. (2018). A review on chemiresistive room temperature gas sensors based on metal oxide nanostructures, graphene and 2D transition metal dichalcogenides. Microchim. Acta.

[2] Kohl D. (2001). Function and applications of gas sensors. J. Phys. D Appl. Phys..

[3] Varghese S.S., Lonkar S., Singh K., Swaminathan S., Abdala A. (2015). Recent advances in graphene based gas sensors. Sens. Actuators B.

[4] Zhang J., Qin Z., Zeng D., Xie C. (2017). Metal-oxide-semiconductor based gas sensors: Screening, preparation, and integration. Phys. Chem. Chem. Phys..

[5] Doujaiji B., Al-Tawfiq J.A. (2010). Hydrogen sulfide exposure in an adult male. Ann. Saudi Med..

[6] Belley R., Bernard N., Côté M., Paquet F., Poitras J. (2005). Hyperbaric oxygen therapy in the management of two cases of hydrogen sulfide toxicity from liquid manure. Can. J. Emerg. Med..

[7] Knight L.D., Presnell S.E. (2005). Death by sewer gas: Case report of a double fatality and review of the literature. Am. J. Forensic Med. Pathol..

[8] Eun S., Reinhart D.R., Cooper C.D., Townsend T.G., Faour A. (2007). Hydrogen sulfide flux measurements from construction and demolition debris (C&D) landfills. Waste Manag..

[9] Panza D., Belgiorno V. (2010). Hydrogen sulphide removal from landfill gas. Process Saf. Environ. Prot..

[10] Xu Q., Townsend T. (2014). Factors affecting temporal H_2_S emission at construction and demolition (C&D) debris landfills. Chemosphere.

[11] Cheng Z., Sun Z., Zhu S., Lou Z., Zhu N., Feng L. (2019). The identification and health risk assessment of odor emissions from waste landfilling and composting. Sci. Total Environ..

[12] Wong Y.C., Ang B.C., Haseeb A., Baharuddin A.A., Wong Y.H. (2019). Conducting polymers as chemiresistive gas sensing materials: A review. J. Electrochem. Soc..

[13] Amírola J., Rodríguez A., Castañer L., Santos J., Gutiérrez J., Horrillo M. (2005). Micromachined silicon microcantilevers for gas sensing applications with capacitive read-out. Sens. Actuator B.

[14] Sedlak P., Sikula J., Majzner J., Vrnata M., Fitl P., Kopecky D., Vyslouzil F., Handel P.H. (2012). Adsorption–desorption noise in QCM gas sensors. Sens. Actuators B.

[15] Manera M.G., Ferreiro-Vila E., García-Martín J.M., Cebollada A., García-Martín A., Giancane G., Valli L., Rella R. (2013). Enhanced magneto-optical SPR platform for amine sensing based on Zn porphyrin dimers. Sens. Actuators B.

[16] Kang Y., Yu F., Zhang L., Wang W., Chen L., Li Y. (2021). Review of ZnO-based nanomaterials in gas sensors. Solid State Ion..

[17] Hsu C.-L., Chang L.-F., Hsueh T.-J. (2017). Light-activated humidity and gas sensing by ZnO nanowires grown on LED at room temperature. Sens. Actuators B.

[18] Diep V.M., Armani A.M. (2016). Flexible light-emitting nanocomposite based on ZnO nanotetrapods. Nano Lett..

[19] Zhang B., Li M., Song Z., Kan H., Yu H., Liu Q., Zhang G., Liu H. (2017). Sensitive H_2_S gas sensors employing colloidal zinc oxide quantum dots. Sens. Actuators B.

[20] Zhang J., Zhao B., Pan Z., Gu M., Punnoose A. (2015). Synthesis of ZnO nanoparticles with controlled shapes, sizes, aggregations, and surface complex compounds for tuning or switching the photoluminescence. Cryst. Growth Des..

[21] Lupan O., Postica V., Gröttrup J., Mishra A.K., De Leeuw N.H., Carreira J.F., Rodrigues J., Ben Sedrine N., Correia M.R., Monteiro T. (2017). Hybridization of zinc oxide tetrapods for selective gas sensing applications. ACS Appl. Mater. Interfaces.

[22] Çolak H., Karaköse E. (2019). Synthesis and characterization of different dopant (Ge, Nd, W)-doped ZnO nanorods and their CO_2_ gas sensing applications. Sens. Actuators B.

[23] Navaneethan M., Patil V., Ponnusamy S., Muthamizhchelvan C., Kawasaki S., Patil P., Hayakawa Y. (2018). Sensitivity enhancement of ammonia gas sensor based on Ag/ZnO flower and nanoellipsoids at low temperature. Sens. Actuators B.

[24] Wei W., Zhao J., Shi S., Lin H., Mao Z., Zhang F., Qu F. (2020). Boosting ppb-level triethylamine sensing of ZnO: Adjusting proportions of electron donor defects. J. Mater. Chem. C.

[25] Ghosh A., Zhang C., Shi S., Zhang H. (2019). High temperature CO_2_ sensing and its cross-sensitivity towards H_2_ and CO gas using calcium doped ZnO thin film coated langasite SAW sensor. Sens. Actuators B.

[26] Gupta S.P., Pawbake A.S., Sathe B.R., Late D.J., Walke P.S. (2019). Superior humidity sensor and photodetector of mesoporous ZnO nanosheets at room temperature. Sens. Actuators B.

[27] Wang J., Xia Y., Zhao H., Wang G., Xiang L., Xu J., Komarneni S. (2017). Oxygen defects-mediated Z-scheme charge separation in g-C_3_N_4_/ZnO photocatalysts for enhanced visible-light degradation of 4-chlorophenol and hydrogen evolution. Appl. Catal. B Environ..

[28] Patil P., Gaikwad G., Patil D., Naik J. (2016). Synthesis of 1-D ZnO nanorods and polypyrrole/1-D ZnO nanocomposites for photocatalysis and gas sensor applications. Bull. Mater. Sci..

[29] Xu J., Pan Q., Qin J. (2000). Sensing characteristics of double layer film of ZnO. Sens. Actuators B.

[30] Geng X., Zhang C., Debliquy M. (2016). Cadmium sulfide activated zinc oxide coatings deposited by liquid plasma spray for room temperature nitrogen dioxide detection under visible light illumination. Ceram. Int..

[31] Zhu L., Zeng W. (2017). A novel coral rock-like ZnO and its gas sensing. Mater. Lett..

[32] Zhu L., Li Y., Zeng W. (2018). Hydrothermal synthesis of hierarchical flower-like ZnO nanostructure and its enhanced ethanol gas-sensing properties. Appl. Surf. Sci..

[33] Korotcenkov G., Cho B. (2012). The role of grain size on the thermal instability of nanostructured metal oxides used in gas sensor applications and approaches for grain-size stabilization. Prog. Cryst. Growth Charact. Mater..

[34] Park S., An S., Mun Y., Lee C. (2013). UV-enhanced NO_2_ gas sensing properties of SnO_2_-core/ZnO-shell nanowires at room temperature. ACS Appl. Mater. Interfaces.

[35] Abdullah A.M., Aziz S.B., Saeed S.R. (2021). Structural and electrical properties of polyvinyl alcohol (PVA): Methyl cellulose (MC) based solid polymer blend electrolytes inserted with sodium iodide (NaI) salt. Arab. J. Chem..

[36] Ali A., Alzamly A., Greish Y.E., Bakiro M., Nguyen H.L., Mahmoud S.T. (2021). A Highly Sensitive and Flexible Metal–Organic Framework Polymer-Based H_2_S Gas Sensor. ACS Omega.

[37] Allam M., Ayesh A.I., Mohsin M.A., Haik Y. (2013). Physical properties of PVA doped with algal glycerol. J. Appl. Polym. Sci..

[38] Abu-Hani A.F., Mahmoud S.T., Awwad F., Ayesh A.I. (2017). Design, fabrication, and characterization of portable gas sensors based on spinel ferrite nanoparticles embedded in organic membranes. Sens. Actuators B.

[39] Singh P., Kumar A., Kaushal A., Kaur D., Pandey A., Goyal R. (2008). In situ high temperature XRD studies of ZnO nanopowder prepared via cost effective ultrasonic mist chemical vapour deposition. Bull. Mater. Sci..

[40] Ristić M., Musić S., Ivanda M., Popović S. (2005). Sol–gel synthesis and characterization of nanocrystalline ZnO powders. J. Alloys Compd..

[41] Mashrai A., Khanam H., Aljawfi R.N. (2017). Biological synthesis of ZnO nanoparticles using C. albicans and studying their catalytic performance in the synthesis of steroidal pyrazolines. Arab. J. Chem..

[42] Roy A.S., Gupta S., Sindhu S., Parveen A., Ramamurthy P.C. (2013). Dielectric properties of novel PVA/ZnO hybrid nanocomposite films. Compos. Part B Eng..

[43] Rawool C.R., Srivastava A.K. (2019). A dual template imprinted polymer modified electrochemical sensor based on Cu metal organic framework/mesoporous carbon for highly sensitive and selective recognition of rifampicin and isoniazid. Sens. Actuators B.

[44] Ali A., AlTakroori H.H., Greish Y.E., Alzamly A., Siddig L.A., Qamhieh N., Mahmoud S.T. (2022). Flexible Cu3 (HHTP) 2 MOF Membranes for Gas Sensing Application at Room Temperature. Nanomaterials.

[45] Jayarambabu N., Kumari B.S., Rao K.V., Prabhu Y. (2014). Germination and growth characteristics of mungbean seeds (*Vigna radiata* L.) affected by synthesized zinc oxide nanoparticles. Int. J. Curr. Eng. Technol..

[46] Ghosh S., Adak D., Bhattacharyya R., Mukherjee N. (2017). ZnO/γ-Fe_2_O_3_ charge transfer interface toward highly selective H_2_S sensing at a low operating temperature of 30 °C. ACS Sens..

[47] Kolhe P.S., Shinde A.B., Kulkarni S., Maiti N., Koinkar P.M., Sonawane K.M. (2018). Gas sensing performance of Al doped ZnO thin film for H_2_S detection. J. Alloys Compd..

[48] Zhang D., Fan X., Hao X., Dong G. (2019). Facile fabrication of polyaniline nanocapsule modified zinc oxide hexagonal microdiscs for H_2_S gas sensing applications. Ind. Eng. Chem. Res..

[49] Yu Z., Gao J., Xu L., Liu T., Liu Y., Wang X., Suo H., Zhao C. (2020). Fabrication of Lettuce-Like ZnO Gas Sensor with Enhanced H_2_S Gas Sensitivity. Crystals.

[50] Shewale P.S., Yun K.-S. (2020). Synthesis and characterization of Cu-dped ZnO/RGO nanocomposites for room-temperature H_2_S gas sensor. J. Alloys Compd..

[51] Ding P., Xu D., Dong N., Chen Y., Xu P., Zheng D., Li X. (2020). A high-sensitivity H_2_S gas sensor based on optimized ZnO-ZnS nano-heterojunction sensing material. Chin. Chem. Lett..

[52] Wang X., Li S., Xie L., Li X., Lin D., Zhu Z. (2020). Low-temperature and highly sensitivity H_2_S gas sensor based on ZnO/CuO composite derived from bimetal metal-organic frameworks. Ceram. Int..

[53] Narayana A., Bhat S.A., Fathima A., Lokesh S., Surya S.G., Yelamaggad C. (2020). Green and low-cost synthesis of zinc oxide nanoparticles and their application in transistor-based carbon monoxide sensing. RSC Adv..

[54] Kumar M., Bhatt V., Abhyankar A.C., Yun J.-H., Jeong H.-J. (2020). Multifunctional dumbbell-shaped ZnO based temperature-dependent UV photodetection and selective H_2_ gas detection. Int. J. Hydrogen Energy.

[55] Wang P., Dong T., Jia C., Yang P. (2019). Ultraselective acetone-gas sensor based ZnO flowers functionalized by Au nanoparticle loading on certain facet. Sens. Actuators B.

[56] Zhao S., Shen Y., Yan X., Zhou P., Yin Y., Lu R., Han C., Cui B., Wei D. (2019). Complex-surfactant-assisted hydrothermal synthesis of one-dimensional ZnO nanorods for high-performance ethanol gas sensor. Sens. Actuators B.

[57] Yuan H., Aljneibi S.A.A.A., Yuan J., Wang Y., Liu H., Fang J., Tang C., Yan X., Cai H., Gu Y. (2019). ZnO nanosheets abundant in oxygen vacancies derived from metal-organic frameworks for ppb-level gas sensing. Adv. Mater..

[58] Wang X., Wang Y., Tian F., Liang H., Wang K., Zhao X., Lu Z., Jiang K., Yang L., Lou X. (2015). From the surface reaction control to gas-diffusion control: The synthesis of hierarchical porous SnO_2_ microspheres and their gas-sensing mechanism. J. Phys. Chem. C.

